# (+)-Usnic acid modulates the Nrf2-ARE pathway in FaDu hypopharyngeal carcinoma cells

**DOI:** 10.1007/s11010-021-04092-7

**Published:** 2021-02-26

**Authors:** Violetta Krajka-Kuźniak, Jarosław Paluszczak, Robert Kleszcz, Wanda Baer-Dubowska

**Affiliations:** Department of Pharmaceutical Biochemistry, Poznan University of Medical Sciences, Poznan, Poland

**Keywords:** FaDu cells, Nrf2, (+)-usnic acid, Arctigenin, Bergenin, Xanthohumol

## Abstract

Naturally occurring phytochemicals of different origin and structure, arctigenin, bergenin, usnic acid and xanthohumol, were shown to affect Nrf2 pathway in the context of various diseases, but their effect on this pathway in cancer cells was not extensively investigated. This study aimed to evaluate the effect of these compounds on Nrf2 expression and activation in hypopharyngeal FaDu squamous cell carcinoma cells. FaDu cells were treated with 2 or 10 μM arctigenin, bergenin, (+)-usnic acid or xanthohumol for 24 h. While arctigenin, bergenin, and xanthohumol did not affect either Nrf2 expression or activation, (+)-usnic acid treatment increased its transcript level and increased the nuclear/cytosol Nrf2 protein ratio—the measure of Nrf2 pathway activation. Consequently, (+)-usnic acid enhanced the transcription and translation of Nrf2 target genes: *NQO1*, *SOD,* and to a lesser extent, *GSTP*. The treatment of FaDu cells with (+)-usnic acid decreased both GSK-3β transcript and protein level, indicating its possible involvement in Nrf2 activation. All the tested compounds decreased *Bax* mRNA but did not change the level of *Bax *protein. (+)-Usnic acid tended to increase the percentage of early apoptotic cells and LC3 protein, autophagy marker. Significant induction of *p53* also was observed after treatment with (+)-usnic acid. In summary, the results of this study indicate that low concentrations of (+)-usnic acid activate Nrf2 transcription factor, most probably as a result of ROS accumulation, but do not lead to FaDu hypopharyngeal carcinoma cells death.

## Introduction

Head and neck squamous cell carcinoma (HNSCC) is the sixth leading cancer in humans. The survival rates in HNSCC patients remain poor, especially in more advanced disease stages when the risk of recurrence is higher. Naturally occurring phytochemicals may support conventional therapy, making it more effective. There are various sources of phytochemicals with chemopreventive potential and their chemical structures show high diversity.

Arctigenin is a natural lignan compound extracted from the seeds of *Arctium lappa L.* (*Asteraceae*) and possesses various biological activities, including anti-oxidant, anti-inflammatory and anti-proliferative action, which were shown in several cancer cells, including gastric, breast and ovarian cancer cells [1, 2]. Bergenin is the principal constituent of the well-known medicinal plant *Saxifraga ligulata Wall.* (*Saxifragaceae*)*.* Its possible anti-cancer activity was shown in colorectal cancer and cervical cancer cells [3, 4]. Xanthohumol is the major prenylated flavonoid present in the hop plant *Humulus lupulus L.* (*Cannabinaceae*) and a common beer ingredient. This biologically active compound also possesses anti-proliferative and cytotoxic activities, shown in colon and hepatocellular carcinoma cells [5, 6].

Usnic acid is one of the most studied secondary metabolites of lichen species (first isolation in 1844). Growing evidence suggests that usnic acid has anti-tumor, anti-oxidative, anti-inflammatory and other activities in various cancer cells. It was recently shown that usnic acid induces cell cycle arrest and autophagy, exerts anti-proliferative and apoptotic effects by modulating the expression of apoptosis-related proteins in gastric neoplastic cells, and has a better anti-tumor effect than 5-fluorouracil in a tumor xenograft model [7]. Two enantiomers of usnic acid occur in nature, but only (+)-usnic acid is easily accessible on the commercial market, and it usually shows slightly higher biological activity [8].

One of these phenolic compounds’ possible targets is nuclear factor erythroid 2-related factor (Nrf2), which binds to the anti-oxidant response element (ARE). The activation of this pathway by reactive oxygen species (ROS) or electrophiles triggers the expression of genes encoding cytoprotective enzymes such as glutathione S-transferases (GST), NAD(P)H:quinone oxidoreductase (NQO1) and superoxide dismutase (SOD). On the other hand, constitutive activation of this pathway in cancer cells may lead to radio- and chemoresistance. Nrf2 is degraded dynamically under normal conditions by the ubiquitin–proteasome system, keeping the expression of its downstream genes at low basal levels. Upon exposure to stress signals, the rate of Nrf2 degradation decreases, while the activation and subsequent accumulation of Nrf2 in the nucleus increases, stimulating the expression of target cytoprotective enzyme systems [9]. The major repressor of Nrf2 is Kelch-like ECH-associated protein-1 (Keap1), which sequesters Nrf2 in the cytoplasm. In response to stress signals, Nrf2 is released from the complex with Keap1 and may translocate to the nucleus [10]. An important role in Nrf2 regulation is played also by glycogen synthase kinase-3 (GSK-3), which phosphorylates serine residues within the DSGIS motif of Nrf2 and promotes its ubiquitination [11]. Moreover, it was shown that Nrf2 may be negatively regulated by tumor suppressor p53, which induces cell cycle arrest and/or apoptosis [12].

While the modulation of the Nrf2–ARE pathway by naturally occurring compounds was extensively studied in several normal and cancer cells, there is a paucity of data on such phenomena in HNSCC cells. This study aimed to investigate the influence of arctigenin, bergenin, (+)-usnic acid and xanthohumol on the expression and activation of Nrf2-ARE pathway in human hypopharyngeal squamous cell carcinoma FaDu cells. The expression of *TP53* and *Bax* genes was also evaluated to assess the potential interactions between p53 and Nrf2 proteins.

## Materials and methods

**Fig. 1:**
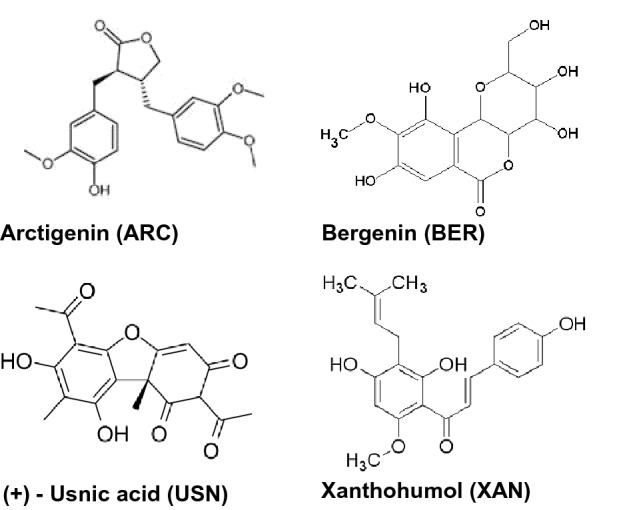
The chemical structure of arctigenin, bergenin, (+)-usnic acid and xanthohumol

Arctigenin (ARC), bergenin (BER), (+)-usnic acid (USN), and xanthohumol (XAN) were obtained from Sigma-Aldrich, USA, and their chemical structure is presented in Fig. 1.

### Cell culture and viability assay

The human FaDu hypopharyngeal carcinoma cell line was purchased from ATCC. Cells were grown in Dulbecco’s modified eagle’s medium (DMEM, Biowest SAS, France) with the addition of 10% FBS (EURx, Poland) and 1% antibiotics solution (penicillin and streptomycin) (Sigma-Aldrich, USA) at 37 °C in 95% humidified and 5% CO_2_ atmosphere. The effect of the tested compounds on the viability of FaDu cells was assessed by the MTT assay, according to a standard protocol. Cells were seeded in 96-well plates (10^4^ per well), and after 24 h of pre-incubation in DMEM supplemented with 5% FBS and antibiotics, the tested compounds were added to the culture medium in various concentrations, and the cells were further incubated for 24 h. Then, cells were washed with PBS buffer, and incubated for 4 h in the presence of fresh medium containing the MTT salt (0.5 mg/ml). Afterward, acidic isopropanol was added into wells in order to dissolve formazan crystals, and absorbance was measured at 570 and 690 nm. All the experiments were repeated three times, with at least four measurements per assay.

For the evaluation of the effect of the tested compounds on the assessed parameters, 2 × 10^6^ cells were seeded in 100 mm culture dishes and, after 24 h of pre-incubation in DMEM containing 5% FBS, cells were treated with either 2 or 10 μM arctigenin, bergenin, (+)-usnic acid or xanthohumol for 24 h. Control cells were treated with the vehicle only (DMSO).

### Isolation of total RNA and cDNA synthesis

Total RNA was isolated using Universal RNA Purification Kit (EURx, Poland) and subsequently subjected to reverse transcription using RevertAid First Strand cDNA Synthesis Kit (Thermo Fisher Scientific, USA), according to the manufacturer’s protocol.

### Quantitative real-time PCR

For quantitative real-time PCR analyses, the Maxima SYBR Green Kit (Fermentas, USA) and a BioRad Chromo4 thermal cycler were used. All qPCR reactions were run in triplicate. The protocol started with 5 min enzyme activation at 95 °C, followed by 40 cycles consisting of 95 °C for 15 s, 54 °C for 20 s and 72 °C for 40 s, and final elongation at 72 °C for 5 min. Melting curve analysis was used for product specificity verification. The estimation of the expression of *TBP* (*TATA box binding protein*) and *PBGD* (*porphobilinogen deaminase*) was used for data normalization. Primer sequences were described previously [6].

### Preparation of cytosolic and nuclear fractions

Subcellular extracts were prepared using the Nuclear/Cytosol Fractionation Kit (BioVision, USA) according to the manufacturer’s protocol. Protein concentration was assessed and the samples were stored at − 80 °C until further analysis.

### Western blot assay

The content of Nrf2, SOD, GSTP, NQO1, GSK-3β, Bax, p53, caspase-3, and LC3 in cellular extracts was assessed using the Western blot technique. Cytosolic (Nrf2, SOD, GSTP, NQO1, GSK-3β, P-GSK-3β, Bax, p53, caspase-3, and LC3) or nuclear (Nrf2, P-Nrf2) extracts were separated on 7.5% SDS-PAGE gels (Bio-Rad, USA) and transferred onto the nitrocellulose membrane. After blocking with 10% skimmed milk, the membranes were incubated with primary polyclonal antibodies directed against Nrf2, SOD, GSTP, NQO1, GSK-3β, P-GSK-3β, Bax, p53, caspase-3, and LC3 (Santa Cruz Biotechnology, USA) and P-Nrf2 (Abcam, Cambridge, UK). β-actin or lamin served as a loading control. After washing, the membranes were probed with alkaline phosphatase-labeled secondary antibodies (anti-rabbit IgG, anti-mouse IgG, anti-goat IgG, Santa Cruz Biotechnology, USA) and stained using the BCIP/NBT AP conjugate substrate kit (Bio-Rad, USA). The quantity one software was used to determine the amount of the immunoreactive products and the values were calculated as relative absorbance units (RQ) per mg protein.

### Nrf2 binding assay

Nrf2 activation in FaDu cells was assessed by enzymatic immunoassay (Nrf2 transcription factor Elisa Assay Kit, Active Motif, Belgium) according to the manufacturer’s instructions. Activated Nrf2 was evaluated based on the amount of Nrf2 contained in the oligonucleotide-binding complex. Oligonucleotides containing the ARE consensus sequence for Nrf2 binding (5′-GTCACAGTGACTCAGCAGAATCTG-3′) were immobilized on microplates as bait. Nuclear fractions were incubated with oligonucleotides for 1 h and then wells were washed and binding was detected using specific primary antibodies against Nrf2 and secondary antibodies conjugated with HRP. The amount of ARE-bound Nrf2 was measured by colorimetric readout at 450 nm.

### Cell cycle distribution

The analysis of the cell cycle distribution was performed using the Muse® Cell Cycle Kit (Merck, Germany) according to the manufacturer’s protocol. Briefly, FaDu cells (3 × 10^5^ per well) were seeded in 6-well plates, pre-incubated for 24 h, and further grown for 24 h in the presence of (+)-usnic acid. Topotecan (50 and 100 nM) served as a positive control of cell cycle arrest. Subsequently, cells were harvested by trypsinization, washed with PBS buffer, fixed in ice-cold 70% ethanol, and frozen at − 20 °C. After overnight storage, fixed cells were washed with PBS buffer, stained with propidium iodide in the presence of RNase A, and subjected to 30 min incubation at room temperature in the dark. The fluorescence signal was analyzed by flow cytometry on Muse® cell analyzer, and data were evaluated using Muse® 1.5 analysis software.

### Apoptosis evaluation

The externalization of phosphatidylserine is one of the best-known apoptosis markers and it can be analyzed by Annexin V staining. Additionally, the 7-Aminoactinomycin D (7-AAD) staining enables discrimination between early and late apoptotic cells. Thus, the Muse® Annexin V & Dead Cell Kit (Merck, Germany) was used for apoptosis evaluation according to the manufacturer’s protocol. Briefly, FaDu cells (3 × 10^5^ per well) were seeded in 6-well plates, pre-incubated for 24 h, and further grown for 24 h in the presence of the ( +)-usnic acid. Topotecan (200 nM) treated cells were used as a positive control of apoptosis induction. After the incubation, cells were harvested by trypsinization, resuspended in fresh medium, stained with Annexin V and 7-AAD solution, subjected to 20 min incubation at room temperature in the dark, and analyzed by flow cytometry on Muse® cell analyzer. Data were evaluated using Muse® 1.5 analysis software.

### Statistical analysis

Statistical analysis was performed using the GraphPad Instat version 3.10 (GraphPad Software, San Diego, USA). The data are presented as means ± SEM. To assess the significance of the evaluated parameters’ changes, one-way ANOVA with Dunnett’s post hoc test was performed with the significance level of *p* < 0.05 and *p* < 0.01.

## Results

### The effect of the chemicals on FaDu cells viability

**Fig. 2:**
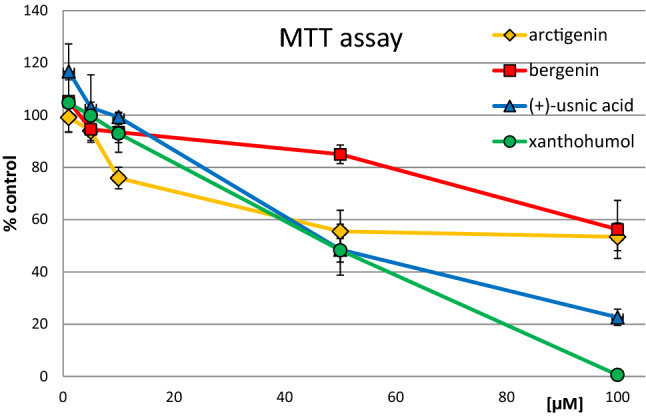
The effect of the tested chemicals on the viability of FaDu cells. Mean values ± SEM from three independent experiments are shown

The effect of the tested compounds on the viability of FaDu cells was assessed using the MTT assay. The results are shown in Fig. 2. Xanthohumol exerted the strongest cytotoxic activity, followed by (+)-usnic acid (IC_50_ values ~ 50 µM). Interestingly, arctigenin was more potent in reducing cell viability than the other compounds at low concentrations (10 µM), however, these differences were not seen at higher concentrations. The lowest cytotoxicity was exerted by bergenin, which reduced cell viability by ~ 40% at the highest concentration (100 µM). To assess these chemicals' modulatory activity, the sub-toxic concentrations of 2 and 10 µM were applied.

### The effect of the chemicals on the expression and activation of Nrf2

**Fig. 3:**
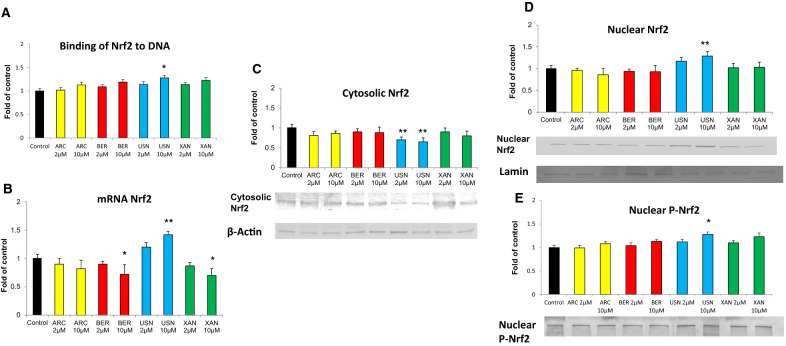
The effect of arctigenin (ARC), bergenin (BER), (+)-usnic acid (USN) and xanthohumol (XAN) on the binding of Nrf2 to DNA (**a**), *Nrf2* transcript level (**b**), Nrf2 translocation from the cytosol (**c**) to the nucleus (**d**) and P-Nrf2 level (**e**) after 24 h incubation. The level of each transcript was calculated in relation to cells treated with the vehicle, where expression was equal 1. Representative Western immunoblots are presented below the graphs. The values were calculated as a relative change in protein level compared to control cells (expression equals 1). Mean values ± SEM from three independent experiments are shown. Asterisk above bars denotes statistically significant changes from DMSO treated (control) cells, *p < 0.05 and **p < 0.01

Quantitative analysis revealed a significant increase (by ~ 20–42%) in the amount of *Nrf2* transcript in FaDu cells as the result of treatment with (+)-usnic acid (Fig. 3). The other tested chemicals, particularly bergenin and xanthohumol at the higher concentration, tended to reduce *Nrf2* transcript level. Nrf2 may induce target gene expression only after its translocation into the nucleus. To investigate the translocation of Nrf2 upon compound treatment, the level of Nrf2 in the cytosolic and nuclear cell fractions was evaluated by the Western blot assay. As shown in Fig. 3, the Nrf2 protein level was decreased in the cytosol and concomitantly increased in the nucleus as the result of treatment with (+)-usnic acid only.

The treatment with this compound also increased the level of nuclear P-Nrf2 due to its phosphorylation at Ser 40. This effect was dose-dependent. To confirm that nuclear Nrf2 can affect gene expression, its ability to interact with ARE consensus sequence was evaluated. Nrf2 activation was measured in terms of the amount of Nrf2 present in the nuclear extract, which was able to bind the oligonucleotide complex containing the ARE consensus site. The results are shown in Fig. 3. (+)-Usnic acid increased the binding of Nrf2 to ARE by ~ 28% compared to the control group. All these data pointed to Nrf2 activation upon the action of (+)-usnic acid.

**Fig. 4:**
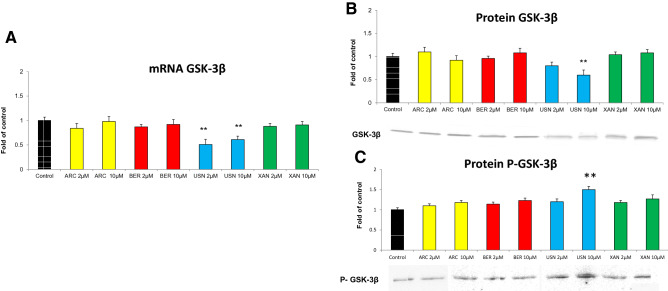
The effect of arctigenin (ARC), bergenin (BER), (+)-usnic acid (USN) and xanthohumol (XAN) on *GSK-3β* transcript (**a**), GSK-3β protein (**b**) and P- GSK-3β level (**c**). The level of each transcript was calculated in relation to cells treated with the vehicle, where expression was equal 1. Representative Western immunoblots are presented below the graphs. The values were calculated as a relative change in protein level compared to control cells (expression equals 1). Mean values ± SEM from three independent experiments are shown. Asterisk above bars denotes statistically significant changes from DMSO treated (control) cells, **p < 0.01

Since the activation of Nrf2 might be affected by GSK-3β, its gene expression was also evaluated. As shown in Fig. 4, the treatment of FaDu cells with (+)-usnic acid reduced both GSK-3β transcript and protein level but elevated the content of its phosphorylated form, P-GSK-3β.

### The effect of the chemicals on the expression of Nrf2 target genes

**Fig. 5:**
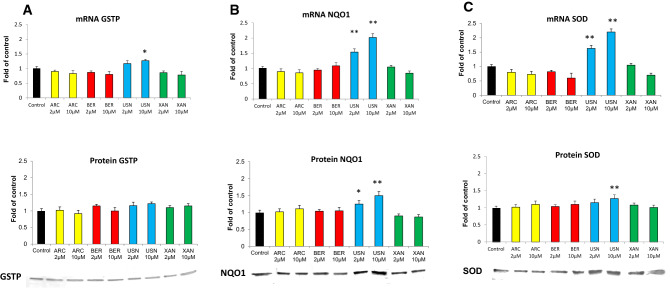
The effect of arctigenin (ARC), bergenin (BER), (+)-usnic acid (USN) and xanthohumol (XAN) on the expression of *GSTP* (**a**), *NQO1* (**b**) and *SOD* (**c**). The level of each transcript was calculated in relation to cells treated with the vehicle where expression was equal 1. Representative Western immunoblots are presented below the graphs. The values were calculated as a relative change in protein level compared to control cells (expression equals 1). Mean values ± SEM from three independent experiments are shown. Asterisk above bars denotes statistically significant changes from DMSO treated (control) cells, *p < 0.05 and **p < 0.01

Figure 5 presents the effect of the tested chemicals on the expression of *GSTP, NQO1,* and *SOD* genes*.* In concert with the increased expression and activation of Nrf2, the treatment of FaDu cells with (+)-usnic acid increased both the transcript and protein levels of both of the evaluated genes encoding phase II enzymes—GSTP and NQO1 (Fig. 5a, b) and also of the anti-oxidant enzyme SOD (Fig. 5c).

The most significant increase in mRNA and protein levels as a result of treatment with (+)-usnic acid was observed in the case of NQO1 (100 and 50%, respectively, at the higher dose). The *SOD* transcript was increased by ~ 120%, but SOD protein was less affected (Fig. 5c).

### The effect of the chemicals on the expression of p53 and Bax proteins

**Fig. 6:**
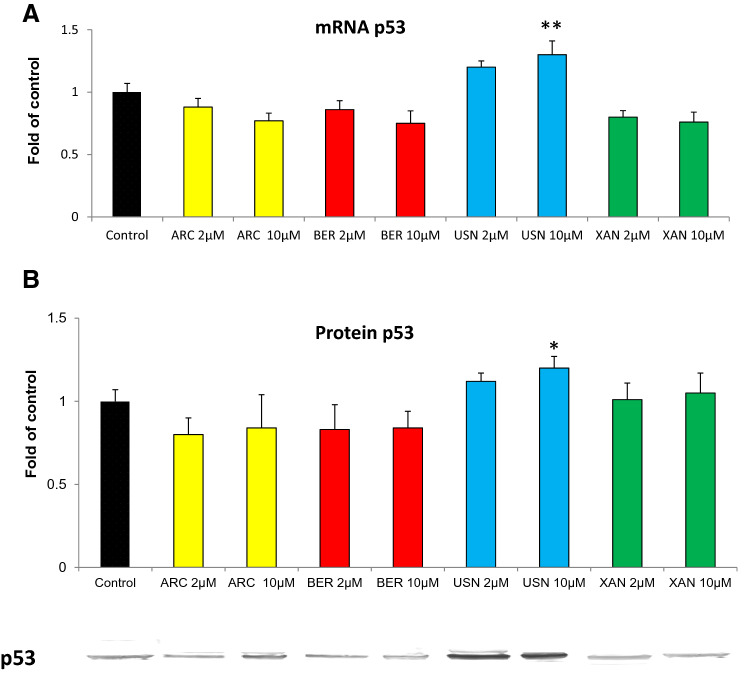
The effect of arctigenin (ARC), bergenin (BER), (+)-usnic acid (USN) and xanthohumol (XAN) on *TP53* transcript (**a**) and p53 protein level (**b**). Transcript level was calculated in relation to cells treated with the vehicle where expression was equal 1. Representative Western immunoblot is presented below the graph. The values were calculated as a relative change in protein level compared to control cells (expression equals 1). Mean values ± SEM from three independent experiments are shown. Asterisk above bars denotes statistically significant changes from DMSO treated (control) cells, *p < 0.05 and **p < 0.01

**Fig. 7:**
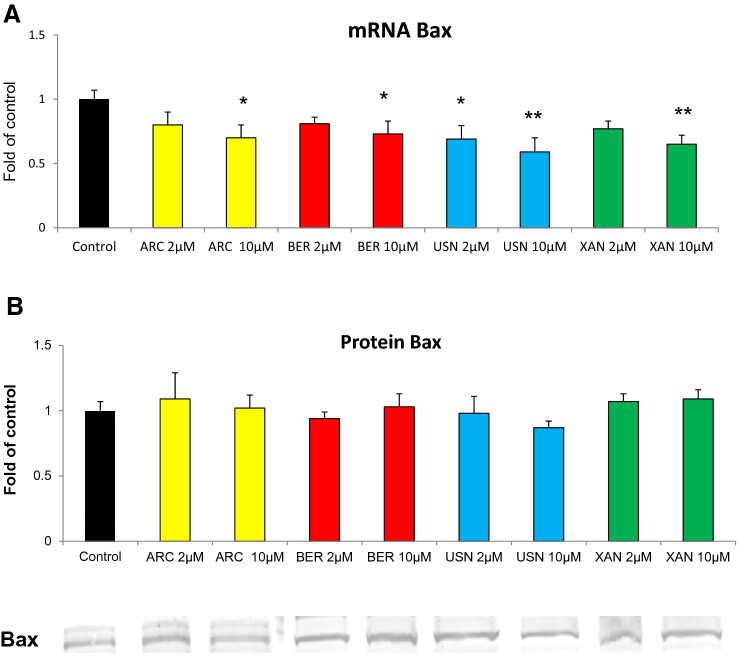
The effect of arctigenin (ARC), bergenin (BER), (+)-usnic acid (USN) and xanthohumol (XAN) on *Bax* transcript (**a**) and Bax protein level (**b**). Transcript level was calculated in relation to cells treated with the vehicle where expression was equal 1. Representative Western immunoblot is presented below the graph. The values were calculated as a relative change in protein level compared to control cells (expression equals 1). Mean values ± SEM from three independent experiments are shown. Asterisk above bars denotes statistically significant changes from DMSO treated (control) cells, *p < 0.05 and **p < 0.01

The most potent modulator of the expression of Nrf2 and its target genes, (+)-usnic acid, also increased *TP53* gene transcript and p53 protein levels, which were not affected by the other tested phytochemicals (Fig. 6). All these compounds at the lower dose tended to reduce the level of *Bax* transcript but did not affect its protein level, although a slight decrease in Bax protein level was observed as the result of treatment with (+)-usnic acid (Fig. 7).

### *The effect of *(+*)-usnic acid on the cell cycle distribution, apoptosis, and autophagy markers*

**Fig. 8:**
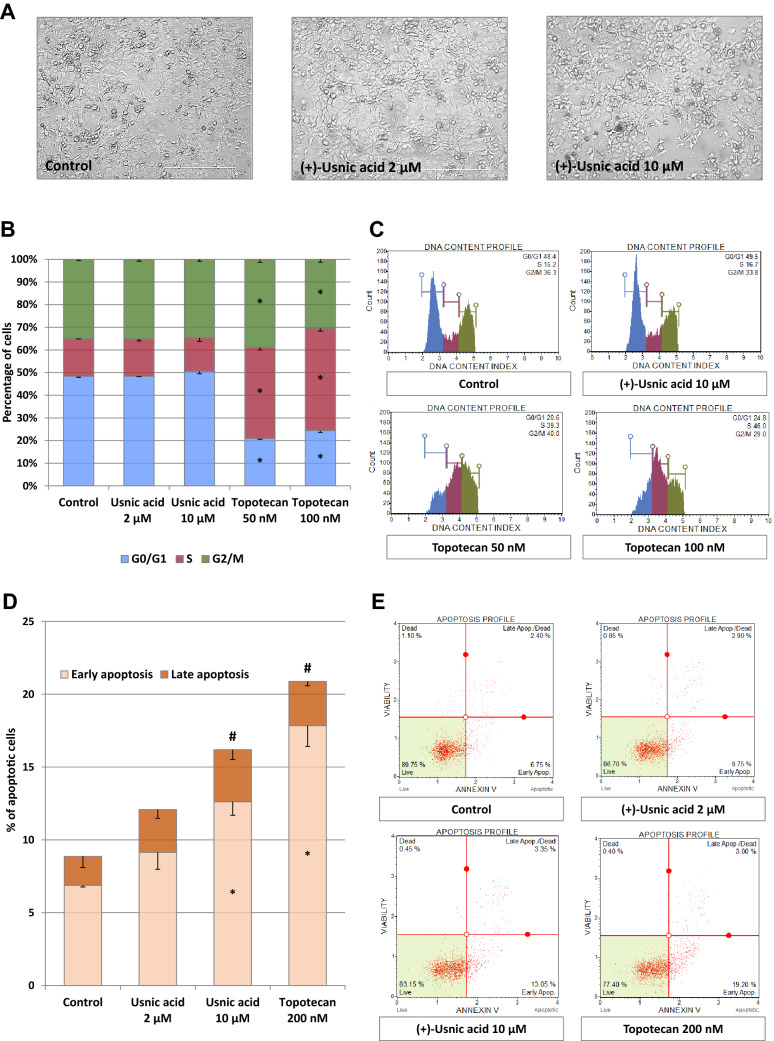
The effect of (+)-usnic acid on FaDu cells morphology (**a**), cell cycle distribution (**b, c**) and apoptosis (**d, e**). Representative images of FaDu cells were taken after 24 h incubation with ( +)-usnic acid or vehicle using JuLI FL microscope (NanoEntek, South Korea) (**a**). Cell cycle distribution was analyzed by flow cytometry after staining with propidium iodide (**b**). Topotecan (50 and 100 nM) was used as a positive control. Representative plots are presented (**c**). Apoptosis was evaluated by flow cytometry after staining with Annexin-V and 7-Aminoactinomycin D (**d**). Topotecan (200 nM) was used as a positive control. Representative plots are presented (**e**). Mean values ± SEM from three independent experiments are shown. Asterisk inside bars denotes statistically significant changes from DMSO treated (control) cells, *p < 0.05. Hash (#) above bars denotes statistically significant changes of the total number of apoptotic cells, *p* < 0.05

**Fig. 9:**
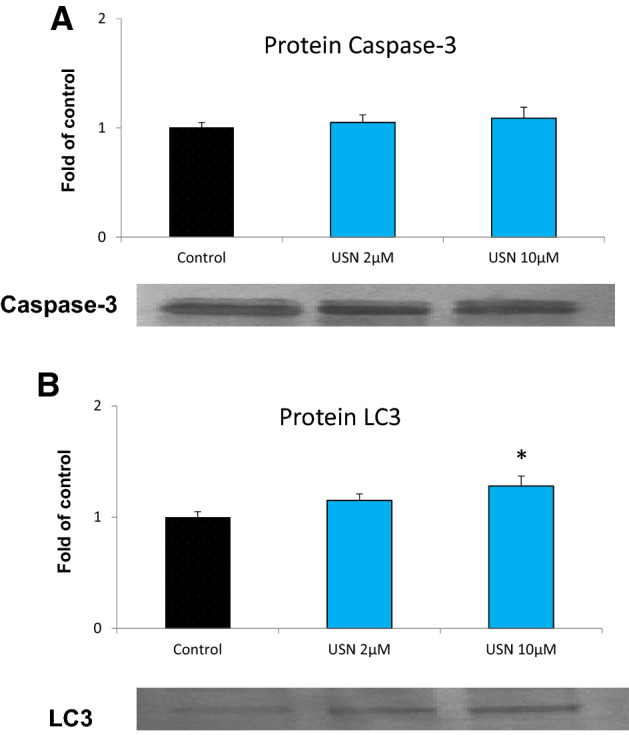
The effect of (+)-usnic acid on caspase-3 (**a**) and LC3 protein level (**b**). Representative Western immunoblots are presented below the graphs. The values were calculated as a relative change in protein level compared to control cells (expression equals 1). Mean values ± SEM from three independent experiments are shown. Asterisk above bars denotes statistically significant changes from DMSO treated (control) cells, *p < 0.05

To find out whether the modulation of Nrf2 activity by (+)-usnic acid may affect cellular functions, the effect on cell cycle distribution and the markers of apoptosis and autophagy was evaluated. As shown in Fig. 8a, cell morphology was not significantly changed upon incubation with (+)-usnic acid. Similarly, treatment with this chemical did not affect cells distribution across the cell cycle (Fig. 8b, c). On the other hand, topotecan, which was used as a reference compound, caused cells accumulation in the S and G2/M phases, according to expectations. Importantly, (+)-usnic acid slightly increased the percentage of early apoptotic cells, but only at the higher concentration (Fig. 8d, e). We can assume that this process was at the initial step because (+)-usnic acid was unable to significantly change the level of the pro-apoptotic caspase-3 protein (Fig. 9). However, the level of LC3 protein, the marker of autophagy was increased due to treatment with (+)-usnic acid in higher tested dose, suggesting induction of autophagy in the cells.

## Discussion

The Nrf2-ARE signaling pathway plays a key role in response to oxidative stress and DNA damaging electrophiles. Many phytochemicals can modify this pathway by affecting Nrf2 expression or activation.

Naturally occurring compounds of different origin and structure, which were the subject of our current study, were shown to affect Nrf2 pathway in the context of various diseases. In this regard, the attenuation of oxidative stress by arctigenin via the activation of Nrf2 signaling pathway was associated with its anti-arrhythmic role in ischemia/reperfusion injury [13]. This compound also modulated Nrf2-ARE axis in rat astrocyte cells [14]. Bergenin was shown to inhibit glucose-induced extracellular matrix (ECM) production in glomerular mesangial cells through the downregulation of oxidative stress via the mTOR/β-TrcP/Nrf2 pathway [15]. Our earlier study showed that xanthohumol increased the expression and led to the activation of Nrf2 in immortalized normal THLE-2 hepatocytes and hepatocellular carcinoma HepG2 cells [6]. Moreover, in a human intervention trial, xanthohumol caused a significant reduction in DNA damage and a clear induction of α-GST (43%), suggesting the involvement of the Keap1-Nrf2 pathway [16].

None of the above-mentioned compounds affected Nrf2 expression or activation in human FaDu hypopharyngeal squamous cell carcinoma cells in this study. However, the treatment of these cells with (+)-usnic acid for 24 h increased both the transcription of *Nrf2* gene and the translocation of Nrf2 protein from the cytosol into the nucleus. Moreover, the activation of Nrf2 resulted in the increased expression of its target genes*, GSTP, NQO1,* and *SOD*.

FaDu cells, similarly to other cell lines derived from HNSCC, were used in several studies as a model to assess the effect of potential chemotherapeutic agents on cell proliferation/growth and induction of apoptosis [17]. However, the data on Nrf2 expression and activation in these cells have not been available so far. Recently, the Nrf2 expression and translocation into the nucleus of FaDu cells was described as a result of nutritional stress [18].

**Scheme 1:**
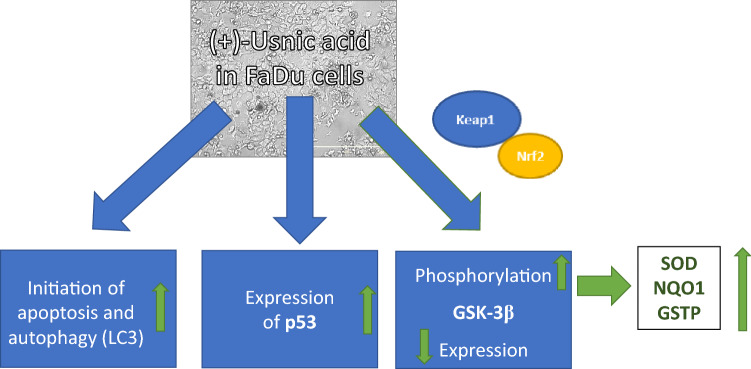
Schematic representation of the results summary

Therefore, our study is one of the first which pointed to the activation of the Nrf2 transcription factor in these cells and its possible modulation by naturally occurring compounds, namely (+)-usnic acid (Scheme 1). This chemical has been widely used as an additive to many products. However, many studies demonstrated that its high doses are hepatotoxic [7]. That is why earlier studies concentrated on the involvement of the Nrf2 pathway in usnic acid toxicity in hepatic cells. In this regard, Chen et al. [19] demonstrated that the Nrf2-dependent adaptive defense response was activated during exposure to usnic acid for 6 h, while S-phase cell cycle arrest, DNA damage, accumulation of ROS, and glutathione depletion were induced with prolonged (24 h) treatment in HepG2 cells. They also showed the direct involvement of Nrf2 in usnic acid-induced cytotoxicity by silencing the expression of the *Nrf2* gene. Overall, these data indicated that the activation of Nrf2 signaling pathway is important in usnic acid-induced DNA damage and cytotoxicity in human hepatocellular carcinoma cells.

In our study, FaDu cells were exposed to (+)-usnic acid for 24 h, thus it is difficult to evaluate the direct role of Nrf2 in its cytotoxicity. Moreover, although the concentrations of (+)-usnic acid which were used in the abovementioned study partly overlapped with the concentrations applied in our study, the most pronounced effect was observed at concentrations higher than 10 µM. The increase in the expression of SOD and NQO1 found in our study may also suggest that Nrf2 expression and activation is an adaptive defense response against ROS accumulation and glutathione depletion induced by (+)-usnic acid also in FaDu cells. Qi et al. [20] recently showed that (+)-usnic acid, at the concentrations range of 10–40 µM, induced apoptosis in lung squamous cell carcinoma (LSCC) cells via disrupting the mitochondrial respiratory chain and inhibiting Nrf2 expression at mRNA and protein levels. Consequently, in contrast to our results, the NQO1 transcript was also reduced in LSCC cells. It is worth noting that usnic acid, at the concentration of 10 µM, reduced the LSCC cells’ viability by 25%. Thus, the effect of usnic acid on Nrf2 and apoptosis might strongly depend on the cancer cell type. Chen et al. [21] calculated human plasma concentration of usnic acid after the intake of a dietary supplement at the recommended daily dose of 100–500 mg. They postulated that after taking such doses of usnic acid, its plasma concentration could reach 7.4–37 µM. Thus, at least the higher concentration of usnic acid applied in our study is clinically relevant.

Nrf2 stability is regulated by Keap1 protein, which targets Nrf2 to its proteasomal degradation and also by GSK-3β, which inhibits Nrf2 function through its phosphorylation [11]. (+)-Usnic acid decreased both GSK-3β transcript and protein levels in FaDu cells. Since the inhibition of Nrf2 function is related to GSK-3β pro-apoptotic effect [22] we can speculate that reduced expression of GSK-3β may be linked with reduced pro-apoptotic Bax protein level in FaDu cells observed as the result of treatment with (+)-usnic acid.

On the other hand, (+)-usnic acid increased the expression of *TP53* gene in these cells. In contrast, the other phytochemicals evaluated in this study tended to reduce it. *TP53* is one of the most important genes in human cancer. It appears that p53 protein is critical for tumor suppression not during the acute responses to cellular stress, which is characterized by extensive apoptosis, but for the killing or silencing of the cancer initiating cells that have acquired oncogenic lesions driving the neoplastic transformation [23]. Moreover, p53 provides a major barrier to tumor progression and metastasis. Missense mutations in the *TP53* gene are extremely widespread in human cancers and give rise to mutant p53 proteins that lose tumor suppressive activities, and some of which exert trans-dominant repression over the wild-type counterpart [24]. Therefore, assuming that increased expression of the wild-type *TP53* gene occurs in cancer cells such as FaDu cells after treatment with (+)-usnic acid, can suggest that (+)-usnic acid may diminish the neoplastic potential of these cells. The increased expression of *TP53* upon usnic acid 6 h exposure was also described in human ovarian carcinoma cells A2780 [25]. The authors suggested that although p53 is not crucial in cell death signaling induction by usnic acid, it may partly affect the overall outcome.

Interestingly, the other phytochemicals tested in this study, i.e. arctigenin, bergenin and xanthohumol induced apoptosis in several cancer cells [26–28]. However, all these compounds, similarly as (+)-usnic acid, particularly at lower concentration (2 µM) reduced the level of *Bax* gene transcript but did not affect its protein in FaDu cells. There are many processes between transcription and translation, which affect the protein level. Moreover, the half-life of different proteins can vary from minutes to days, whereas the degradation rate of mRNA would fall within a much tighter range, 2–7 h for mammalian mRNAs versus 48 h for protein [29]. Therefore, many processes may contribute to discrepancy between *Bax* gene mRNA and protein level observed in this study. It also has to be noted that (+)-usnic acid treatment at higher (10 µM) concentration increased the level of LC3 protein, the marker of autophagy, suggesting induction of this process in FaDu cells.

In summary, the results of this study indicate that low concentrations of (+)-usnic acid induce the expression and activate Nrf2 transcription factor most probably as the result of ROS accumulation, but do not lead to hypopharyngeal carcinoma FaDu cells death. More detailed studies are necessary to explain these observations better and to verify their possible application to HNSCC therapy.
